# High molecular diagnostic yields and novel phenotypic expansions involving syndromic anorectal malformations

**DOI:** 10.1038/s41431-022-01272-x

**Published:** 2023-01-04

**Authors:** Heiko Reutter

**Affiliations:** 1 Division of Neonatology and Pediatric Intensive Care, Department of Pediatrics and Adolescent Medicine, Friedrich-Alexander University of Erlangen-Nürnberg, Erlangen, Germany; 2 Institute of Human Genetics, Friedrich-Alexander University of Erlangen-Nürnberg, Erlangen, Germany

**Keywords:** Anal diseases, Disease genetics

## Classification of congenital anorectal malformations (ARMs)

In this issue if EJHG, Belanger Deloge et al. [1] present the diagnostic yield of exome analysis among individuals with anorectal malformations (ARM). ARM comprise congenital malformations of the hindgut and represent the most common malformations of the lower digestive tract. The overall prevalence ranges from two to five per 10,000 births [2]. Mild phenotypes as cutaneous perineal fistula may easily be missed, especially among affected females, which may partly explain male/female ratios being 1.2–1.6 [2]. In 2005, the Krickenbeck Conference on ARM developed standards for an “International Classification system” describing up to 10 distinct subtypes ranging from anal stenosis to severe and complex cloacal malformations [3]. All of these ARMs may occur isolated (non-syndromic ARMs), in combination with one or more co-occurring anomalies, or as part of a genetic syndrome (syndromic ARMs). Previous studies found up to 75% of individuals to present with additional anomalies [4]. Most of these co-occurring anomalies belong to the congenital anomaly spectrum of the VATER/VACTERL association. The VATER/VACTERL association refers to the nonrandom co-occurrence of at least three of the following component features: vertebral defects (V), ARMs (A), cardiac defects (C), tracheoesophageal fistula with or without esophageal atresia (TE), renal malformations (R), and limb defects (L) [5]. In accordance with the case classification guidelines for the National Birth Defects Prevention Study [6], individuals with ARM with a chromosomal or single gene disorder, a defined clinical syndrome, mental retardation, and/or dysmorphisms have syndromic ARM. While about 10% of syndromic ARM might be explained by chromosomal disorders, the overall contribution of single gene disorders remains elusive [4]. Until today about 30 known monogenic syndromes have been described with ARM as an inherent phenotypic feature e.g., Baller-Gerold syndrome (#218600, *RECQL4*), Kabuki syndrome (#147920, *KMT2D*), Opitz-Kaveggia syndrome (#305450, *MED12*), Townes-Brocks syndrome 1 (#107480, *SALL1*).

## High diagnostic yield for syndromic ARMs

In their study, Belanger Deloge et al. [1] describe the exome analysis of 130 individuals with ARM, identified in a clinical database of about 17,000 individuals referred for exome analysis. In 45 of these individuals a definitive or probable diagnosis was made (34.6%). Moreover, Belanger Deloge et al. [1] identified eight phenotypic expansions of know genetic syndromes comprising Helmsmoortel-van der Aa syndrome (# 615873, *ADNP*), Bardet-Biedl syndrome 1 (# 209900, *BBS1*), Rubinstein-Taybi syndrome 1 (# 180849, *CREBBP*), Rubinstein-Taybi syndrome 2 (# 613684, *EP300*), Fanconi anemia, complementation group C (# 227645, *FANCC*), Kabuki syndrome 2 (# 300867, *KDM6A*), Luscan-Lumish syndrome (*SETD2*-related disorder) (# 616831, *SETD2*), and Coffin-Siris syndrome 4 (# 614609, *SMARCA4*). These findings suggest that single gene disorders underly a much larger proportion of syndromic ARMs than previously thought.

## ARM in the context of the VATER/VACTERL association

On the contrary, Belanger Deloge et al. [1] suggest that tests designed to identify monogenic etiologies may have lower diagnostic yields in individuals with ARM in the context of the VATER/VACTERL association (22.8% vs 44.1%). The authors suggest that the contribution of epigenetic and environmental factors might play a more important role in the formation of the VATER/VACTERL association than previously thought. However, to date, no consistent environmental risk factor has been identified that could be specifically responsible for the development of the VATER/VACTERL association [7]. In addition, Solomon et al. [5] provided several lines of evidence that dominant single-gene disorders may underlie a certain number of multiply affected families with VATER/VACTERL association, in which environmental risk factors are unlikely to play a significant role.

## Non-coding regions of the genome: fruits that may hang lower than we think?

Hitherto, the search for genetic risk factors for congenital birth defects has mostly focused on the protein-coding genome not encountering the multiplicity of regulatory regions and the respective non-coding RNAs residing in disease loci [8]. One reason, why the hunt for non-coding RNAs or regulatory elements has been carried out with hand brakes applied in regards to the VATER/VACTERL association, might be the difficulties to provide functional proof of the anticipated genetic alterations in embryonic animal models [8]. However, there are several examples that suggest that these regions must not be neglected any longer. De Pontual et al. [9] described hemizygous germline deletions of *MIR17HG*, encoding the miR-17∼92 polycistronic miRNA cluster, to cause Feingold syndrome (# 164280), an autosomal dominant syndrome comprising microcephaly, short stature, and digital anomalies. Interestingly, less penetrant defects within the phenotypic spectrum of Feingold syndrome include learning disabilities of variable degree, esophageal and duodenal atresias (observed in 30–55% of cases), and cardiac and renal malformations, representing several component features of VATER/VACTERL spectrum. Studying the genetic basis of congenital limb malformations, which also belong to the VATER/VACTERL association spectrum, Flötmann et al. [10] identified several disease-causing CNVs that interfered with normal gene regulation by either altering enhancer dosage or changing the architecture of so called topologically associating domains. Finally, Long et al. [11] showed very recently, that upregulation of miR-92a-2-5p are implicated in the formation of ARMs in a rat model.

Hence, what is the hardest of all? What seems easiest to you. To see with your eyes what lies before you - the non-coding genome (Johann Wolfgang von Goethe).
